# Supplementary material for: Identification of photoperiod-induced specific miRNAs in the adrenal glands of Sunite sheep (Ovis aries)
Source: Front Vet Sci. 2022 Jul 22;9:888207. doi: 10.3389/fvets.2022.888207 (PMC9354845; doi:10.3389/fvets.2022.888207)
Supplement: Supplementary file 1 [file Data_Sheet_1.ZIP › Supplementary file/miRDeep2 file.zip!/miRDeep2 file/Novel_Structure/LP42A3/Novel_13.pdf]

Provisional ID	:	NC_019459.2_2732
Score total	:	2.4
Score for star read(s)	:	-1.3
Score for read counts	:	0
Score for mfe	:	2.1
Score for randfold	:	1.6
Score for cons. seed	:	
Total read count	:	66
Mature read count	:	62
Loop read count	:	0
Star read count	:	4

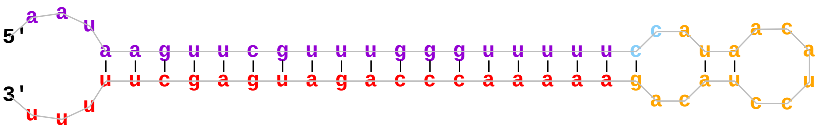

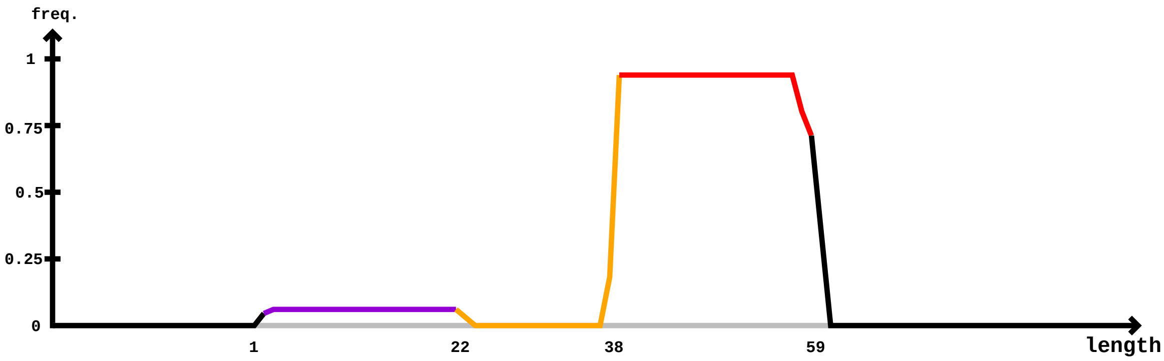


```
                    Star                                Mature
5'- aaauacauguuggaguggccaauaaguucguuuggguuuuuccauaacauccuacagaaaaacccagaugagcuuuuuggccaacccaauacuuuauuuccuuuucggguc  -3'  obs
    aaauacauguuggaguggccaauaaguucguuuggguuuuuccauaacauccuacagaaaaacccagaugagcuuuuuggccaacccaauacuuuauuuccuuuucggguc       exp
    .......((((((..(((((((.(((((((((((((((((((..((......))..))))))))))))))))))).)))))))..))))))........(((....)))..  reads  mm  sample
    ....................aauaaguucguuuggguuuuu......................................................................  3      0   seq
    .....................auaaguucguuuggguuuuu......................................................................  1      0   seq
    ........................................................gaaaaacccagaugaAcuuu...................................  6      1   seq
    ........................................................gaaaaacccagaugaAcuuuu..................................  6      1   seq
    .........................................................aaaaacccagaugaAcuuu...................................  3      1   seq
    .........................................................aaaaacccagaugaAcuuuuu.................................  45     1   seq
    .........................................................aaaaacccagaugaAcuuuuug................................  2      1   seq
```
